# Human Physiology

**Published:** 1839-01

**Authors:** 


Art. VII. — *Human Physiology; illustrated by Engravings.* By Robley Dunglison, m.d. m.a.p.s. &c. *Third Edition, with numerous Additions and Modifications.—Philadelphia*, 1838. In two Vols. 8vo. pp. 1172.

We are happy to believe that the rapid sale of the last edition of this valuable work may be regarded as an indication of the extending taste for sound physiological knowledge in the American schools; and what we then said of its merits will show that we regarded it as deserving the reception it has experienced; since, without claim to originality of thought, it presented the student with a judicious summary of the state of knowledge in most departments of the subject. In preparing for the present edition, Dr. Dunglison has, we are glad to perceive, anticipated the recommendation which we gave in regard to the addition of references; and he has thereby not only added very considerably to the value of his work, but has shown an extent of reading which, we confess, we were not prepared by his former edition to expect. He has also availed himself of the additional materials supplied by the works that have been published in the interval, especially those of Müller and Burdach; so that, as a collection of details on human physiology alone, we do not think that it is surpassed by any other work in our language; and we can recommend it to students in this country as containing much with which they will not be likely to meet elsewhere. Still, however, some parts are a little antiquated; and, in giving the brief results of late enquiries on several topics, we think that the author might have omitted those of older date, which only tend to perplex the reader by their discrepancy. To our own minds, for example, the researches of Dr. Montgomery on the characters of the genuine corpus luteum are perfectly satisfactory; and, by explaining all the facts upon which previous opinions were based, supersede the necessity of reference to them. For, in opposition to such statements as his, founded upon careful and discerning comparison of different structures that have been associated under the same appellation, it is of no avail to bring forwards the vague opinions of Blumenbach or Sir Everard Home. No two authors, however, are ever likely to agree on all departments of a science which involves so many disputed questions as physiology; and it is but fair to say that our opinion of the judicious character of Dr. Dunglison's summary, has been confirmed, in a great majority of instances, by a renewed examination of this edition of his work.

